# BAFF and APRIL expression as an autoimmune signature of membranous nephropathy

**DOI:** 10.18632/oncotarget.23232

**Published:** 2017-12-14

**Authors:** Seung Seok Han, Seung Hee Yang, Hyung Ah Jo, Yun Jung Oh, Minkyoung Park, Joo Young Kim, Hajeong Lee, Jung Pyo Lee, Sang-Ho Lee, Kwon Wook Joo, Chun Soo Lim, Yon Su Kim, Dong Ki Kim

**Affiliations:** ^1^ Department of Internal Medicine, Seoul National University College of Medicine, Seoul, Korea; ^2^ Kidney Research Institute, Seoul National University, Seoul, Korea; ^3^ Department of Internal Medicine, Seoul National University Boramae Medical Center, Seoul, Korea; ^4^ Department of Internal Medicine, College of medicine, Kyung Hee University, Seoul, Korea

**Keywords:** APRIL, autoimmunity, BAFF, B cells, membranous nephropathy

## Abstract

**Background:**

Based on the fact that B-cell activating factor (BAFF) and a proliferation-inducing ligand (APRIL) have a regulatory role in B cell biology, excessive levels of these cytokines can promote autoimmune pathogenesis. However, the expression and implication remain unresolved in cases of membranous nephropathy (MN).

**Results:**

The plasma BAFF levels of the primary MN patients were higher than those of healthy controls but lower than those of secondary MN patients, whereas the APRIL levels were similar between the MN patients and healthy controls. The BAFF levels were higher in relapse cases, whereas the APRIL levels were higher in the patients who did not experience remission compared with the counterpart patients. The ectopic expression of BAFF and APRIL was observed in the glomeruli or circulating B cells of MN patients, and this high expression trend was similar to that of lupus patients.

**Conclusions:**

Expression profile of BAFF and APRIL in MN is similar to that of other autoimmune disease, which affects the kidney outcomes.

**Methods:**

Plasma BAFF and APRIL levels were measured upon kidney biopsy in patients with primary (*n* = 89) and secondary MN (*n* = 13), and the results were compared with the levels in healthy controls (*n* = 111). The kidney outcomes (e.g., remission and relapse) were traced for the median of 3 years. Aberrant expression of the cytokines was evaluated in the kidney and circulating B cells using immunohistochemistry and flow cytometry analyses, respectively.

## BACKGROUND

Membranous nephropathy (MN) is a major cause of nephrotic syndrome, particularly in adults [1], and it ultimately leads to the failure of kidney function [2]. Approximately 75% of MN cases are named as primary or idiopathic disease because their pathophysiology is not well understood. However, recent endeavor has revealed culprit antigens in human podocytes, such as M-type transmembrane phospholipase A_2_ receptor (PLA2R) [3] and thrombospondin type-1 domain-containing 7A (THSD7A) [4], although further exploration of different antigens is needed. These results suggest that MN is an autoimmune disease; however, our understanding has been less focused on other factors influencing autoimmune milieu.

B-cell activating factor (BAFF) and a proliferation-inducing ligand (APRIL) are both tumor necrosis factor-like molecules that share certain receptors that are primarily expressed in B cells [5]. Based on the experimental clues, both of these cytokines release signals to promote the differentiation and longevity of B cells, although certain immune modulating aspects may be different between the two [6]. In addition to these fundamental roles, the cytokines are involved in galvanizing B cells to become self-reactive [7–9], and elevated blood or tissue levels of BAFF and APRIL are frequently observed in various autoimmune diseases [10, 11] and other B cell disorders [12–15]. This issue has driven researchers to explore the role of BAFF and APRIL in MN pathogenesis and translate optimistic or pessimistic results to the clinical use of preexisting inhibitors against BAFF and APRIL [16, 17]. Herein, we analyzed the expression levels of BAFF and APRIL and tracked corresponding kidney outcomes in patients with MN. Additionally, we assessed aberrant expressive features of cytokines, which have also been observed in other autoimmune diseases.

## RESULTS

The baseline characteristics of the enrolled patients are shown in Table 1. The patients underwent kidney biopsies because of proteinuria and the corresponding nephrotic features. The causes of secondary MN were cancer (*n* = 6), hepatitis (*n* = 3), and others, such as Kimura’s disease, interstitial lung disease, hypereosinophilic syndrome, and Castleman’s disease. The patients with primary MN received blockers of the renin-angiotensin-aldosterone system or steroids after diagnosis.

**Table 1:** Baseline characteristics of the study subjects

Parameters	Primary MN (*n* = 89)	Secondary MN (*n* = 13)
Age (years)	57.1 ± 13.81	55.3 ± 12.17
Male (%)	60.7	46.2
Hypertension (%)	49.4	30.8
Diabetes mellitus (%)	25.8	7.7
Laboratory findings		
Serum creatinine (mg/dL)	0.8 (0.66–0.99)	0.9 (0.81–1.16)
Serum albumin (g/dL)	2.8 ± 0.72	2.6 ± 0.80
Serum cholesterol (mg/dL)	263.4 ± 97.58	292.2 ± 114.49
Urine protein / creatinine ratio (g/g)	6.0 (3.02–9.95)	6.7 (2.66–9.19)
eGFR (mL/min/1.73 m^2^)	89.4 ± 24.44	76.7 ± 25.23
Treatment agents (%)		
ACEi / ARB	74.2	46.2
Steroid	58.4	46.2
Cyclosphosphamide	30.3	30.8
Others	23.6	15.4
Pathologic stage (%)		
Stage I	15.7	38.5
Stage II	40.4	15.4
Stage III	38.2	46.2
Stage IV	5.6	0
Tubulointerstitial fibrosis (%)		
None	28.1	7.7
Mild	62.9	76.9
Moderate to severe	9.0	15.4
Plasma BAFF (ng/mL)	0.9 ± 0.26	1.6 ± 1.09
Plasma APRIL (ng/mL)	0 (0–0.53)	0 (0–1.62)

Abbreviations: eGFR, estimated glomerular filtration rate; ACEi, angiotensin-converting-enzyme inhibitor; ARB, angiotensin receptor blocker; BAFF, B-cell activating factor; APRIL, a proliferation-inducing ligand.

### Plasma BAFF and APRIL

**Figure 1:**
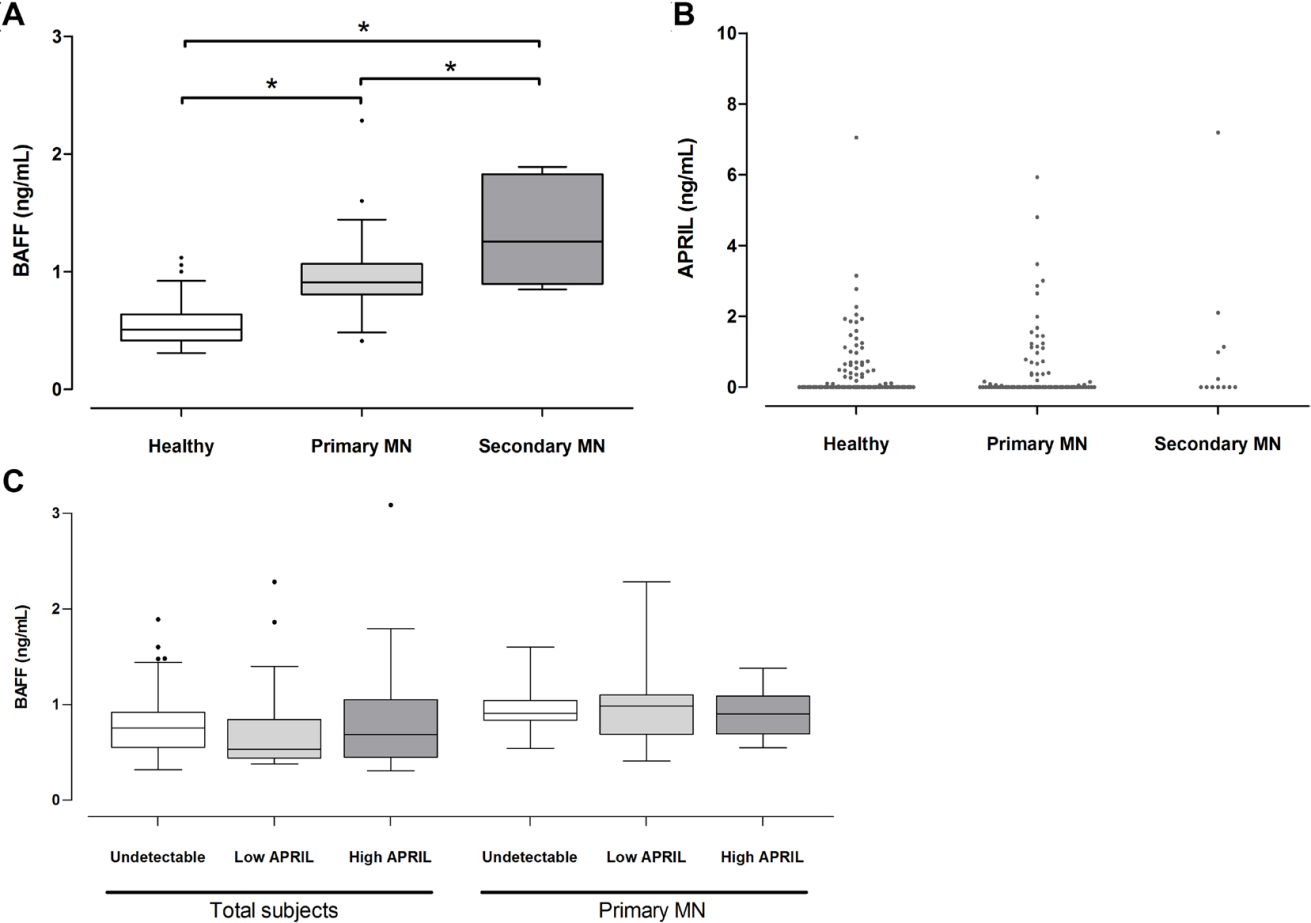
Plasma BAFF and APRIL levels in patients with MN The levels of BAFF (**A**) and APRIL (**B**) were expressed by box-and-whisker and dot plot, respectively, and compared with those of healthy controls or patients with secondary MN. (**C**) When the correlation was evaluated between BAFF and APRIL, the significance was not shown. ^*^P < 0.05.

A comparison results of the plasma cytokine levels showed that the BAFF levels of the primary MN patients (0.9 ± 0.26 ng/mL) were higher than those of the healthy controls (0.5 ± 0.16 ng/mL) (*P* < 0.001) but lower than those of the secondary MN patients (1.6 ± 1.09 ng/mL) (*P* = 0.011) (Figure 1A). For the plasma APRIL, 59.6% and 53.8% of the patients with primary and secondary MN had undetectable levels, whereas 61.3% of the healthy controls had undetectable levels. The median levels did not differ between groups (Figure 1B). The BAFF and APRIL levels were not correlated each other in total subjects or patients with MN (Figure 1C). The expression patterns indicated that BAFF and APRIL presented different relationships with the MN as suggested by previous study of other autoimmune diseases [6].

**Figure 2:**
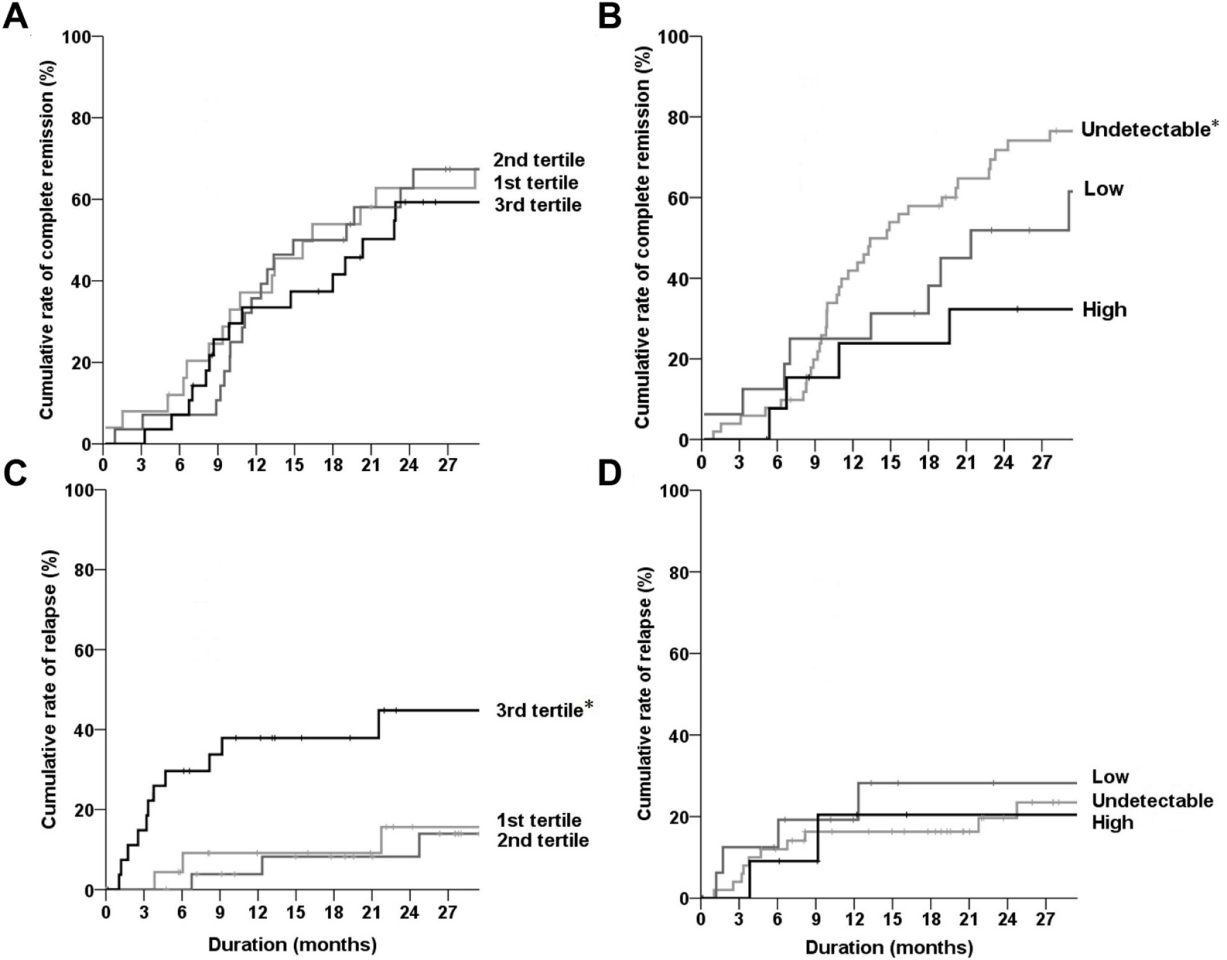
Kaplan–Meier curves of complete remission or relapse according to the plasma levels of BAFF and APRIL (**A**, **B**) Rates of complete remission were compared among the tertiles of BAFF (A) or three APRIL groups (B). (**C**, **D**) Rates of relapse were compared among the tertiles of BAFF (C) or three APRIL groups (D). Black, dark gray, and gray lines represent the 3rd tertile (or high APRIL), the 2nd tertile (or low APRIL), and the 1st tertile (or undetectable APRIL) group of BAFF, respectively. ^*^P < 0.05.

To explore the relationship of BAFF and APRIL with kidney outcomes, the primary MN patients were deliberately divided into three groups based on the distribution of BAFF (tertiles) and APRIL (undetectable and detectable groups including low (<median value), and high (≥median value) groups). The rates of complete remission did not differ among the BAFF groups (Figure 2A); however, higher APRIL levels were observed in patients with less complete remission compared with the counterpart groups (Figure 2B). For the relapse events, the 3rd tertile group of BAFF had a higher rate than other groups (Figure 2C), whereas the APRIL levels did not affect the relapse outcome (Figure 2D). The HR results also followed the same trend as the Kaplan–Meier curves, which did not vary based on other covariate effects (Table 2 for complete remission and Table 3 for relapse).

**Table 2:** Comparison of complete remission rates according to the plasma levels of BAFF and APRIL

			Univariate		Multivariate^*^	
Marker	Group	Level (ng/mL)	HR (95% CI)	*P*	HR (95% CI)	*P*
BAFF	1st tertile (*n* = 29)	0.7 ± 0.12	1 (Reference)		1 (Reference)	
	2nd tertile (*n* = 30)	0.9 ± 0.05	0.96 (0.514–1.785)	0.891	0.98 (0.465–2.066)	0.958
	3rd tertile (*n* = 30)	1.2 ± 0.25	1.02 (0.546–1.905)	0.952	0.80 (0.360–1.770)	0.580
APRIL	Undetectable (*n* = 53)	0	1 (Reference)		1 (Reference)	
	Low (*n* = 18)	0.3 (0.07–0.47)	0.76 (0.399–1.447)	0.403	0.83 (0.347–1.958)	0.662
	High (*n* = 18)	1.6 (1.21–3.13)	0.40 (0.176–0.887)	0.024	0.23 (0.076–0.706)	0.010

^*^Adjusted for age, sex, hypertension, diabetes mellitus, baseline creatinine, albumin, cholesterol, urine protein to creatinine ratio, estimated glomerular filtration rate, the use of angiotensin-converting enzyme inhibitors/angiotensin receptor blockers, steroids, cyclophosphamides or other immunosuppressive agents, pathologic stage, and tubulointerstitial fibrosis.

Abbreviations: BAFF, B-cell activating factor; APRIL, a proliferation-inducing ligand; HR, hazard ratio; CI, confidence interval.

**Table 3:** Hazard ratios for relapse after treatment response according to the levels of BAFF and APRIL

			Univariate		Multivariate^*^	
Marker	Group	Level (ng/mL)	HR (95% CI)	*P*	HR (95% CI)	*P*
BAFF	1st tertile (*n* = 24)	0.7 ± 0.12	1 (Reference)		1 (Reference)	
	2nd tertile (*n* = 27)	0.9 ± 0.06	0.59 (0.136–2.523)	0.472	0.85 (0.151–4.796)	0.851
	3rd tertile (*n* = 28)	1.2 ± 0.26	3.13 (1.016–9.670)	0.047	5.57 (1.394–22.258)	0.015
APRIL	Undetectable (*n* = 50)	0	1 (Reference)		1 (Reference)	
	Low (*n* = 16)	0.3 (0.07–0.60)	1.37 (0.458–4.091)	0.570	0.96 (0.280–3.299)	0.961
	High (*n* = 13)	1.6 (1.23–2.93)	1.08 (0.294–3.967)	0.904	0.99 (0.174–5.645)	0.990

^*^Adjusted for age, sex, hypertension, diabetes mellitus, baseline creatinine, albumin, cholesterol, urine protein to creatinine ratio, estimated glomerular filtration rate, the use of angiotensin-converting enzyme inhibitors/angiotensin receptor blockers, steroids, cyclophosphamides or other immunosuppressive agents, pathologic stage, and tubulointerstitial fibrosis.

Abbreviations: BAFF, B-cell activating factor; APRIL, a proliferation-inducing ligand; HR, hazard ratio; CI, confidence interval.

### Ectopic expression of BAFF and APRIL

**Figure 3:**
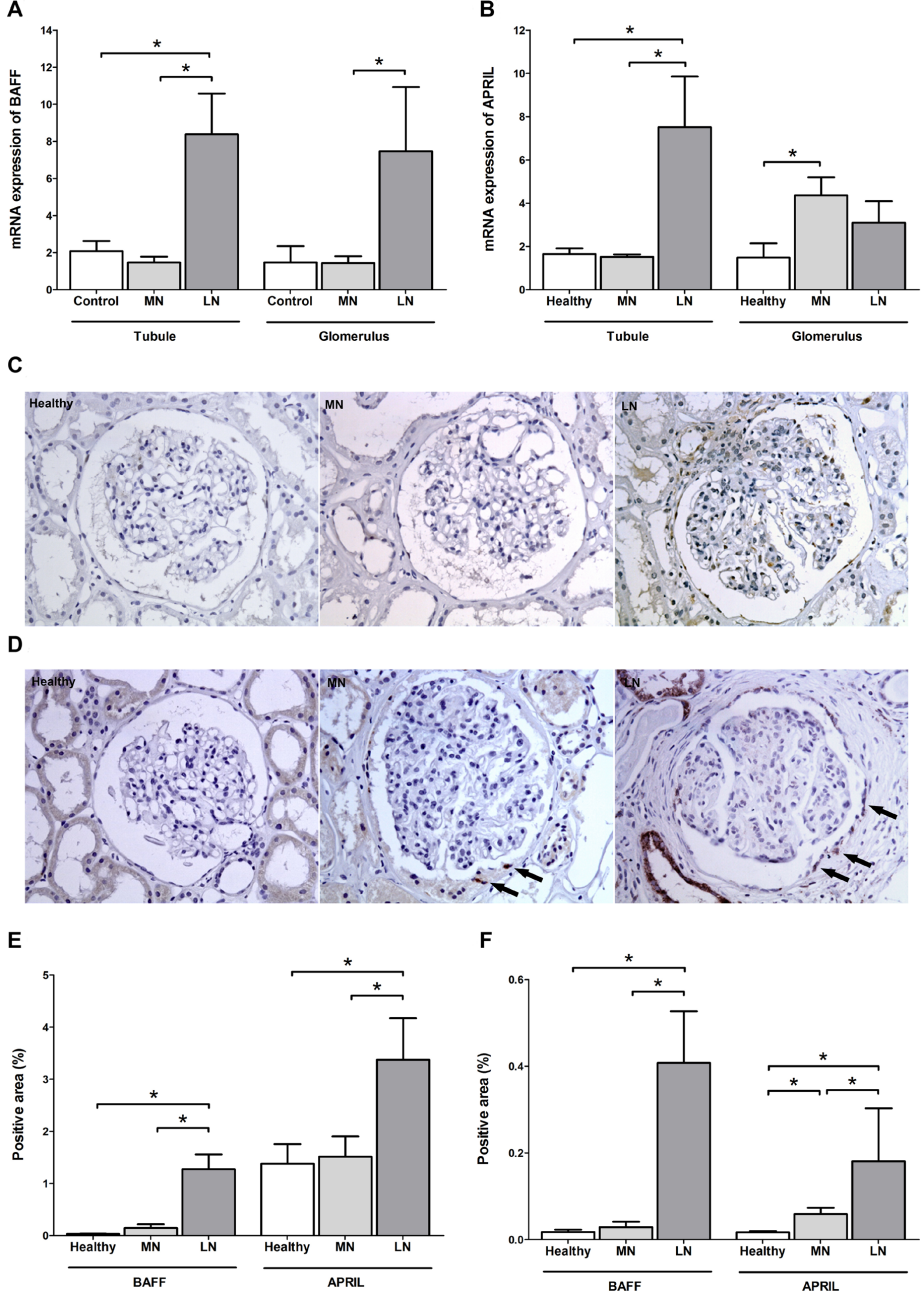
BAFF and APRIL expressions in kidney tissues (**A**, **B**) Comparison in the mRNA expressions of BAFF (A) and APRIL (B) between kidney tissues of healthy controls and patients with MN and lupus nephritis. (**C**, **D**) Representative images of BAFF (C) and APRIL (D) staining from healthy controls and patients with MN or lupus nephritis. Arrows indicates APRIL-positive staining within parietal epithelial cells. (**E**, **F**) Semiquantitative immunostaining scores of BAFF and APRIL in tubulointerstitium (E) and glomeruli (F). LN, lupus nephritis. ^*^P < 0.05.

The main sources of BAFF and APRIL are stromal tissue, monocytes, and T cells [18, 19]; thus, their expression patterns likely affect the crosstalk with pathogenic B cells. A recent study indicated that BAFF and APRIL are highly expressed in the glomeruli and tubulointerstitium of patients with lupus nephritis [20]. In this respect, we investigated the expression profiles in the MN patients (*n* = 28) and compared them with the profiles of healthy controls (*n* = 7) and patients with lupus nephritis (*n* = 13). The relative mRNA quantity of BAFF was similar between the MN patients and healthy controls (Figure 3A). For the APRIL, the glomeruli of the MN patients had higher levels than those of the healthy controls, whereas the tubulointerstitium of the MN patients did not differ with that of healthy controls (Figure 3B). Both the glomeruli and tubulointerstitium of lupus patients had higher trends of mRNA quantities than the tissues of the other groups. The immunohistochemical staining trend was similar to the above results as shown in Figure 3C–3F. Intriguingly, the positive pattern of APRIL in the MN patients was prominent in the parietal epithelial cells (as the arrows in the Figure 3D), which is consistent with the results of a previous report comparing membranous lupus nephritis to the overall mesangial staining pattern of proliferative lupus nephritis [21].

We induced podocyte and parietal epithelial cell injuries by various stimulants *in vitro* to confirm that the expression patterns of BAFF and APRIL were a common feature in other stimulation settings. However, the results did not resemble the mRNA expression and immunohistochemical staining trends of MN based on the observations that the fold change of BAFF or APRIL did not increase under various stimulations except the lipopolysaccharide-treated podocytes (Supplementary Figure 1). Rather, the expression of APRIL in parietal epithelial cells was reduced by certain stimulants such as transforming growth factor-β and angiotensin II. These results indicate that the increased expression of APRIL in patients with MN (especially in parietal epithelial cells) was not a common feature of stimulation settings, but may be distinct characteristics of autoimmune diseases.

**Figure 4:**
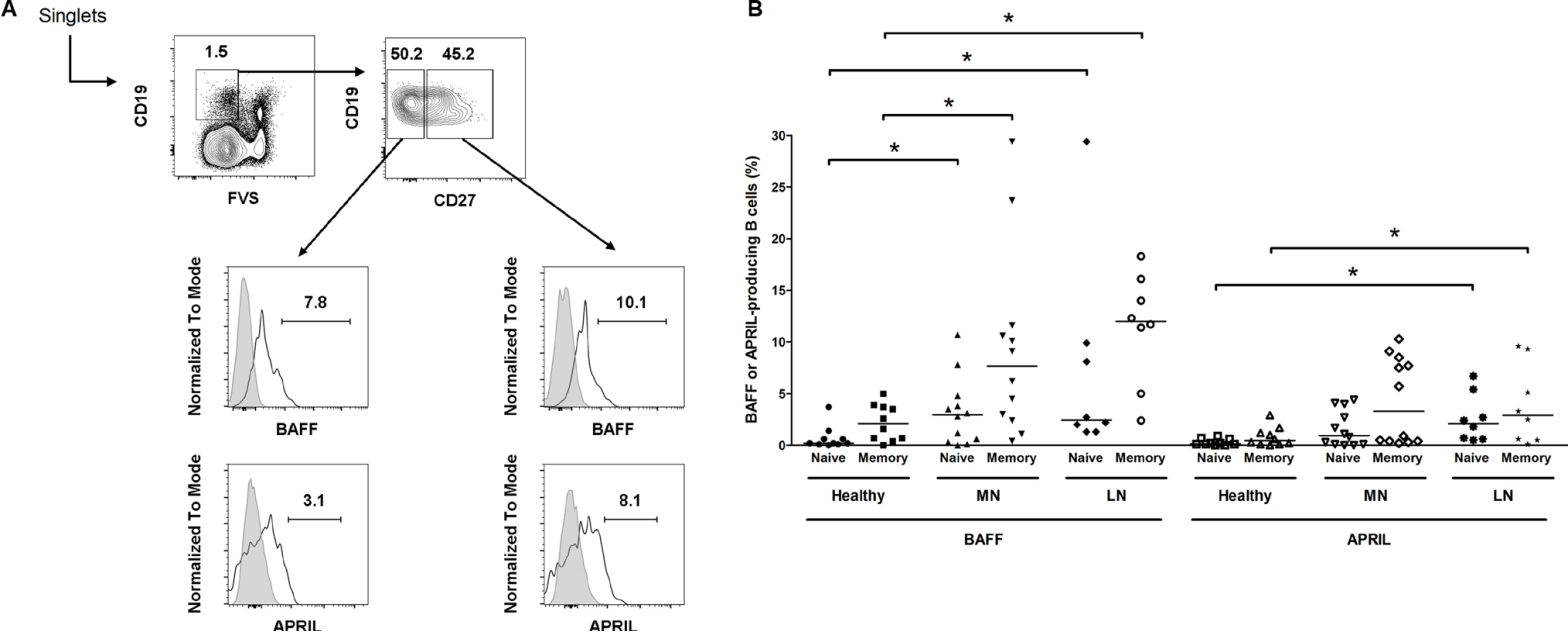
Flow cytometric analysis of BAFF or APRIL-producing B cells among total B cells (**A**) Representative gating strategy for B cells (upper) and histograms of BAFF^+^ and APRIL^+^ B cells (lower). (**B**) Pooled data of healthy controls and patients with MN or lupus nephritis. Lines represent the median levels. FVS, fixable viability stain; LN, lupus nephritis. ^*^P < 0.05.

The abnormal production of BAFF and APRIL from B cells has been documented in patients with systemic lupus nephritis [21, 22] and the corresponding murine models [23]. We questioned whether this phenomenon could also be observed in the blood of patients with MN (*n* = 12) in comparison to healthy controls (*n* = 7) and patients with lupus nephritis (*n* = 6) (Figure 4). When naïve (live CD19^+^ CD27^−^) and memory (live CD19^+^ CD27^+^) B cells were gated, high levels of BAFF-secreting B cells were observed in the MN and lupus nephritis patients were observed compared with the healthy controls. This trend was stronger in the memory B cells than in naïve B cells. However, although the APRIL-secreting cells were high in certain MN patients, the overall differences between the MN patients and healthy controls were not determined. Taken together, the ectopic expression profiles in kidney tissues and peripheral B cells indicate that several distinct characteristics of autoimmune diseases, such as systemic lupus erythematosus, are also observed in MN.

## DISCUSSION

Recent results for podocyte-specific antigens and their corresponding autoantibodies vertically move our understanding of the pathophysiology of MN, which is now identified as an autoimmune disease; however, the systemic immune process of MN has not been investigated in depth. The present work primarily focused on the expression profiles of BAFF and APRIL in patients with MN, and high levels were observed according to the type of cytokine or location. Furthermore, the levels were associated with the clinical outcomes of the patients. These results provide new insights into the roles of BAFF and APRIL in the complex pathophysiology of MN and suggest that these roles are consistent with the pathophysiology of other autoimmune diseases.

The mechanism by which autoantibodies (e.g., anti-PLA2R antibody) are produced in MN has not been determined. The appearance of neoantigens on the surface of podocytes is the first prerequisite; however, this step may not fully account for the pathophysiology because PLA2R, the most well-known antigen, is normally expressed in the podocyte membrane [3]. Although autoimmune perturbations (e.g., antigen processing, such as neoepitopes or post-translational modifications, and antigen speading) could be proposed, the further strides to understand the role of systemic immune system are demanded. The intriguing point is a high recurrence rate of MN after kidney transplantation (up to 40%) [24], which indicates that changes in podocytes or the corresponding antigens alone could not negate the development of MN. Another interesting issue is the cooccurrence of several autoantibodies in MN [25] and the presentation of anti-PLA2R antibodies in secondary MN and other types of nephritis [26], which raises a possibility of common systemic process in autoantibody production.

BAFF and APRIL are autoimmunity-polarizing factors. BAFF-transgenic mice exhibit an autoimmune disease that resembles systemic lupus erythematosus [7]. Although the phenotype of APRIL-transgenic mice is less dramatic than that of BAFF-transgenic mice, the overexpression of APRIL in murine models promotes immune dysregulation via the activity of B and T cells [27]. The preclinical observations are also clinically reflected in several cases of autoimmune diseases [10, 11], including the current cases of MN. Although the present study did not identify the direct roles of BAFF and APRIL, functional evidence, such as the longevity of B cell survival, class switching to immunoglobulin G, and repertoire-selective tolerance [5, 28, 29], indicates that high levels of these cytokines may drive B cells to become pathogenic and produce autoantibodies. Intriguingly, the relationships with kidney outcomes differed between BAFF and APRIL, which suggests that the role of an autoimmune-bearer may vary between the cytokines.

The underlying mechanism may differ between primary and secondary MN. Primary MN is clearly derived from typical autoimmune processes [3, 4], but the mechanisms of secondary MN are undetermined until now. The different immune processes between the two may affect the different plasma levels of BAFF. Further studies are needed to clearly address the present findings.

Certain stromal cells secrete high levels of BAFF and APRIL to promote crosstalk with B cells [30, 31]. High glomerular expressions of APRIL were observed in MN cases than in healthy individuals, and this was not originated from infiltrating inflammatory cells. The cell population responsible for the APRIL expression was the parietal epithelial cells. Active crosstalk between components (podocytes, parietal epithelial cells, endothelial cells, and mesangial cells) should exist [32], and the APRIL in parietal epithelial cells may be the signaling component to others or the response marker after receiving signals from others. Nevertheless, the overall expression intensity of glomeruli was lower than that of lupus nephritis. Considering the low expression in the tubulointerstitium, MN may confer less autoimmune feature with regard to stromal cells, compared with lupus nephritis.

We observed BAFF- or APRIL-secreting B cells in the peripheral blood of the MN patients, and the trend was similar to that of lupus nephritis. These expressions may add up the total pool of cytokines and modify the functions of B cells to favor autoimmune milieu [21, 30]. Previous study documented that *in vitro* activation of murine B cells up-regulated the expressions of BAFF and APRIL and this trend was shown in the lupus murine model [23]. The results may provide the potential role of autocrine activation of pathogenic B cells in MN as well as other autoimmune diseases. Nevertheless, further studies are warranted to understand the role of ectopic expressions particularly in disease outcome.

The fundamental immunosuppressive regimen for treating MN includes steroids, alkylating agents, or calcineurin inhibitors [33, 34]. Despite their reasonable efficiency, other therapeutic options are required because the current regimens can have potential side effects after long-term use. In terms of BAFF and APRIL, two different blocking options are currently available for autoimmune diseases, such as the anti-BAFF monoclonal antibody and a recombinant fusion protein containing a receptor (i.g., transmembrane activator and CAML interactor) fused to the Fc portion of immunoglobulin [16, 17]. Because positive and negative aspects of such treatments have been observed, a variety of trials must be further attempted to achieve a personalized approach in each patient. In this respect, the results presented here may provide a good foundation for future strategies focused on modulating BAFF and APRIL in cases of MN.

The present data inevitably invokes further requirement of data and discussion, because the main focus presented was the correlation and expression profiles. Serial cytokine levels were not examined particularly after immunosuppressive agent use. These limitations should be addressed in an independent cohort to further determine the scientific and clinical role of BAFF and APRIL.

An incomplete understanding of the pathophysiology of MN, particularly in terms of the autoimmune system, has hampered the improvement of clinical outcomes. The present study revealed the expression profiles of BAFF and APRIL in MN, which affected the kidney outcomes. These results may help our understanding of pathophysiology of MN. Nevertheless, more thorough understanding should be obtained by determining the autoimmune features exhibited in cases of MN or examining other various perspectives, and then, can be ultimately applied to the real clinical practice of patients with MN.

## MATERIALS AND METHODS

### Patients and data collection

The study protocol complies with the Declaration of Helsinki and received full approval from the institutional review board at the Seoul National University Hospital (no. 1409-153-616). A total of 102 patients with MN were diagnosed by kidney biopsy from September 2009 and December 2014. Among them, 89 patients did not show evidence of secondary causes (e.g., malignancy and infection) at the time of and after diagnosis; they were diagnosed with primary MN according to the serology-based algorithm [35]; and the other 13 patients were diagnosed with secondary MN. All of the patients provided written informed consent for the donation and use of their specimens, including blood and kidney tissues, in the present study.

The recorded clinical parameters included the following: age, sex, history of hypertension and diabetes mellitus, and the use of therapeutic agents, such as angiotensin-converting-enzyme inhibitor/angiotensin receptor blocker, steroids, and other immunosuppressive agents. The recorded blood parameters were creatinine, albumin, and cholesterol. The glomerular filtration rates were estimated using the Chronic Kidney Disease Epidemiology Collaboration creatinine equation [36]. Proteinuria was quantified using a urine protein to creatinine ratio (uPCR). All of these baseline parameters were assessed when the kidney biopsy was performed for diagnosis. Biopsied slides were reviewed and graded from stage I to IV based on an ultrastructural characterization [37]. Additionally, the lesions of interstitial fibrosis and tubular atrophy were semi-quantitatively divided as follows: none, mild (<25%), and moderate to severe (≥25%). The primary outcome was the first achievement of complete remission, which was defined as <0.2 g/g of uPCR. A relapse event was also recorded when the uPCR re-increased (>3.5 g/g) during a response to the treatment (<3.5 g/g as well as half the baseline uPCR value). Patients were followed up for the median duration of 3 years (maximum 6 years).

### ELISA for BAFF and APRIL

All of the patient plasma was acquired at the time of kidney biopsy and before any treatment given. The plasma BAFF and APRIL levels were then measured using an enzyme-linked immunosorbent assay kit (R&D Systems and eBioscience). Simultaneously, we established control groups to compare the plasma levels of BAFF and APRIL with the MN group. A total of 111 healthy individuals were recruited at the time of health checkup. All of these individuals had a serum creatinine level of less than 1.1 mg/dL, and they did not have proteinuria, hematuria, or a history of comorbidities. Baseline characteristics of these individuals are shown in the Supplementary Table 1.

### Defining MN based on the serology-based approach

MN cases were classified according to the serology-based algorithm [35]. Anti-PLA2R antibody was quantified via the enzyme-linked immunosorbent assay kit (Euroimmun). We defined a negative state as <14 U/mL. In the MN patients with negative titer, staining for PLA2R and THSD7A antigens in the biopsied kidney tissues was performed. Based on this algorithm, 70 and 2 patients were associated with PLA2R and THSD7A, respectively. The others (*n* = 17) were not associated with both antigens.

### Quantitative PCR for BAFF and APRIL

Unfixed biopsy cores were exposed to RNase inhibitor and microdissected into glomerular (including Bowman’s capsule) and tubular specimens. RNA was isolated from each kidney tissue compartment with an RNeasy Micro kit (Qiagen). Subsequently, RNA was converted to cDNA using a kit according to the manufacturer’s protocol (Promega Biotech). The TaqMan gene expression assay was used for human BAFF, APRIL, synaptopodin, and Wilms’ tumor 1, with the endogenous control (glyceraldehyde 3-phosphate dehydrogenase) (Thermo Fisher Scientific). The quantitation of the results was performed by the comparative Ct (2^-(delta)(delta)Ct^) method.

### Immunohistochemistry of kidney tissues

Formalin-fixed paraffin-embedded specimen slides (4 µm-thick section) from biopsied kidney tissues were prepared. After performing a standard deparaffinization technique, the slides were stained with anti-BAFF or anti-APRIL antibody (Novus Biologicals) and then counterstained with hematoxylin. The morphometric parameters for positive BAFF or APRIL were determined using a microscope coupled to a computerized morphometry system (Leica). As negative and positive controls, kidney tissues from healthy individuals and patients with lupus nephritis were used, respectively.

### Isolation and culture of human podocytes and parietal epithelial cells

Primary human podocytes were obtained from the unaffected pole of a nephrectomy specimen of renal cell carcinoma. Kidney cortexes were mechanically dissected and glomerular cells were sieved using optimized media [38] and subsequently isolated using the flow cytometric method with the following markers: podocytes, nephrin^+^CD31^−^; and parietal epithelial cells, nephrin^-^claudin-1^+^ (nephrin: polyclonal, ThermoFisher Scientific; CD31: WM-59, eBioscience; claudin-1: 421203, R&D Systems). The purities of sorted cells were >98%. For the *in vitro* stimulation, the cells were maintained at a concentration of 1 × 10^5^ cells per well in a 6-well plate and fasted for 24 hours, and they were then treated with 2 ng/mL of recombinant transforming growth factor-β, 400 μM of angiotensin II, or 50 ng/mL of lipopolysaccharide for 48 hours.

### Flow cytometry of B cells

Peripheral blood mononuclear cells were obtained using a Ficoll-density gradient. These cells were then stimulated with 50 ng/mL of phorbol 12-myristate 13-acetate, 1 μg/mL of ionomycin, and GolgiStop (BD Biosciences) for 4 hours. The Fc receptor blocking reagent (eBioscience) was initially used in all of the samples, and then sequential surface and intracellular staining were performed with the following antibodies: CD19 (HIB19; BioLegend), CD27 (M-T271; BD Biosciences), BAFF (1D6; eBioscience), and APRIL (REA347; Miltenyi Biotec). A fixable viability stain was applied to exclude dead cells (BD Biosciences). Data were acquired with an LSR Fortessa (BD Biosciences) and were analyzed with FlowJo software (FlowJo LLC).

### Statistical analysis

All of the analyses and calculations were performed using SPSS (version 23.0; IBM) and STATA (version 12.0; StataCorp). Data are presented as the mean ± standard deviation for continuous variables and as proportions for categorical variables. Based on variable distributions determined using histograms, the variables with non-normal distributions are expressed as medians (interquartile ranges). The comparisons were evaluated using the chi-square test for categorical variables, an ANOVA for normally distributed continuous variables (LSD post hoc analysis between two groups), and the Kruskal-Wallis test for non-normally distributed continuous variables (Mann-Whitney *U*-test between two groups). The correlation coefficient between continuous variables was measured using Pearson’s correlation test or a linear regression model. The Cox proportional hazard model with and without covariate adjustments was used to calculate the hazard ratios (HRs) for the outcomes. A *P* value less than 0.05 was considered significant.

## SUPPLEMENTARY MATERIALS FIGURE AND TABLE



## Abbreviations

APRIL: A proliferation-inducing ligand
BAFF: B-cell activating factor
HR: hazard ratio
MN: membranous nephropathy
PLA2R: M-type transmembrane phospholipase A_2_ receptor
THSD7A: thrombospondin type-1 domain-containing 7A
uPCR: urine protein to creatinine ratio
