# Supplementary material for: BAFF and APRIL expression as an autoimmune signature of membranous nephropathy
Source: Oncotarget. 2017 Dec 14;9(3):3292–302. doi: 10.18632/oncotarget.23232 (PMC5790464; doi:10.18632/oncotarget.23232)
Supplement: Supplementary file 1 [file oncotarget-09-3292-s001.pdf]

BAFF and APRIL expression as an autoimmune signature of membranous nephropathy

SUPPLEMENTARY MATERIALS

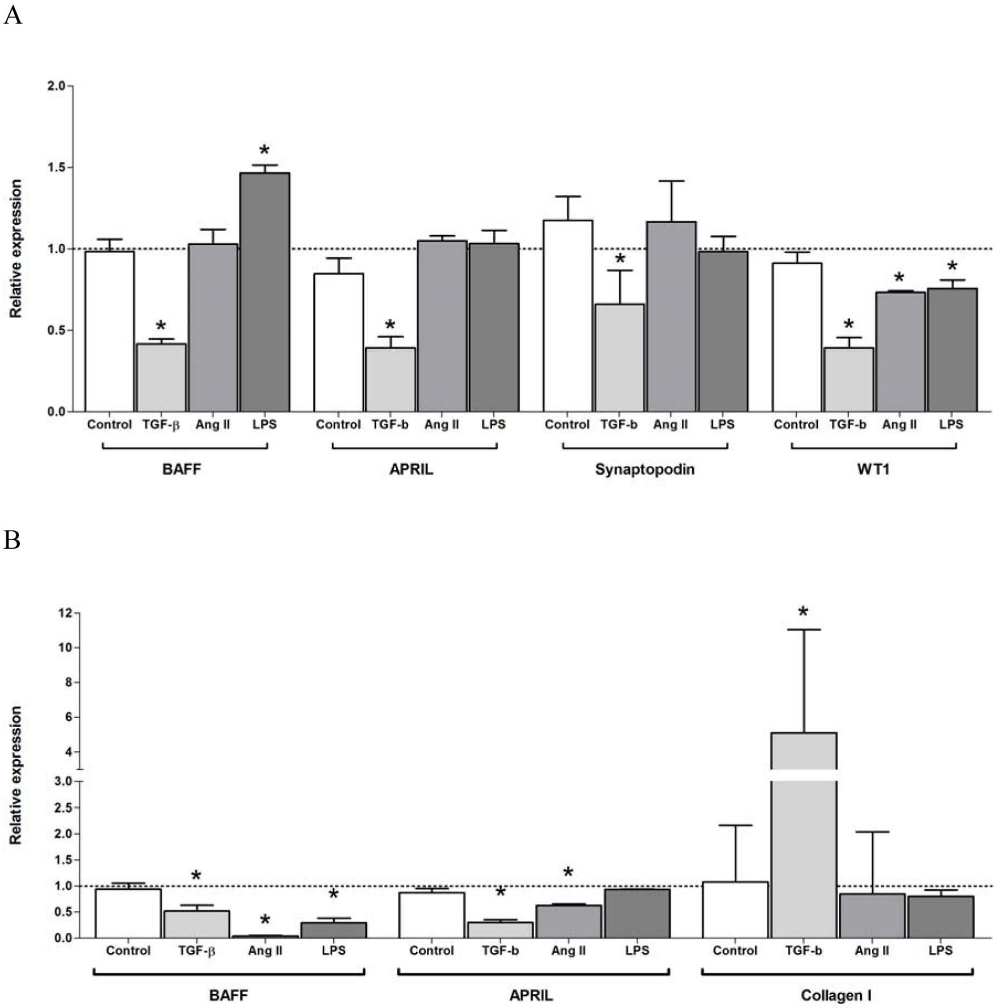

Supplementary Figure 1: mRNA expressions of BAFF and APRIL according to the use of various stimulants. (**A**) mRNA expressions in human primary podocytes. Expressions of synaptopodin and WT1 were also evaluated to confirm the damage of podocytes. (**B**) mRNA expressions in human parietal epithelial cells. Expression of collagen type I was also measured to confirm the fibrosis of parietal epithelial cells. TGF-β, transforming growth factor-β; Ang, angiotensin; LPS, lipopolysaccharide; WT1, Wilms tumor 1. $^{*}P < 0.05$.

Supplementary Table 1: Baseline characteristics of the control group

	Controls for plasma examination (*n* = 111)
Age (years)	28.9 ± 4.7
Male sex (%)	79.8
Hypertension (%)	0
Diabetes mellitus (%)	0
Dyslipidemia (%)	0
Cardiovascular disease (%)	0
Malignancy (%)	0
Serum creatinine (mg/dL)	0.9 ± 0.11
Estimated GFR (mL/min/1.73 m^2)	114.3 ± 9.00
Abnormality in dipstick test (%)	0
Plasma BAFF (ng/mL)	0.5 ± 0.16
Plasma APRIL (ng/mL)	0 (0–0.48)

GFR, estimated glomerular filtration rate; BAFF, B-cell activating factor; APRIL, a proliferation-inducing ligand.
